# Chromosomal copy number based stratification of gastric cancer has added prognostic value to Lauren’s histological classification

**DOI:** 10.1038/s44276-024-00078-2

**Published:** 2024-08-19

**Authors:** H. D. Biesma, T. T. D. Soeratram, H. F. van Essen, J. M. P. Egthuijsen, J. B. Poell, E. van Dijk, E. Meershoek - Klein Kranenbarg, H. H. Hartgrink, C. J. H. van de Velde, M. A. van de Wiel, B. Ylstra, N. C. T. van Grieken

**Affiliations:** 1 Department of Pathology, Cancer Center Amsterdam, Amsterdam University Medical Centers, VU University, Amsterdam, the Netherlands; 2 Department of Otolaryngology / Head and Neck Surgery, Amsterdam University Medical Centers, VU University, Amsterdam, the Netherlands; 3 Department of Surgery, Leiden University Medical Center, Leiden, the Netherlands; 4 Department of Epidemiology and Biostatistics, Amsterdam University Medical Centers, VU University, Amsterdam, the Netherlands

## Abstract

**Background:**

The Cancer Genome Atlas (TCGA) recognizes four molecular subgroups of gastric cancer: Epstein-Barr virus (EBV) positive, microsatellite instable (MSI), genomically stable (GS), and chromosomal instable (CIN). Since a GS/CIN classifier is lacking, alternative markers such as Lauren’s histopathology or CDH1/p53 immunohistochemistry are commonly applied. Here we compared survival of gastric cancer subgroups determined by four methods.

**Methods:**

309 EBV negative and microsatellite stable tumors were included from the Dutch D1/D2 trial and assigned to subgroups by: (i) TCGA’s specific chromosomal copy number aberrations, (ii) genome instability index (GII), (iii) Lauren’s classification, and (iv) CDH1/p53 immunohistochemistry. Subgroups were associated with cancer-related survival (CRS).

**Results:**

Five-year CRS was 42.0% for diffuse and 49.5% for patients with intestinal type tumors, and 57.8% for GS and 41.6% for patients with CIN tumors. Classification by GII or CDH1/p53 IHC did not correlate with CRS. The combination of TCGA and Lauren classifications resulted in four distinct subgroups. Five-year CRS for GS-intestinal (*n* = 24), GS-diffuse (*n* = 57), CIN-intestinal (*n* = 142) and CIN-diffuse (*n* = 86) was 61.4%, 56.5%, 47.6%, and 31.5%, respectively.

**Conclusions:**

TCGA’s GS and CIN subgroups have additional prognostic value to Lauren’s classification in resectable gastric cancer. GS-intestinal, GS-diffuse, CIN-intestinal and CIN-diffuse are suggested stratification variables for future studies.

## Introduction

Gastric cancer is a heterogeneous disease with variable clinical outcome [1]. Over the last decades, this heterogeneity has been addressed by different classification systems [2–9]. The diagnostically most established system is the histology-based Lauren classification that recognizes diffuse and intestinal types of gastric adenocarcinomas as the main two subtypes, of which the latter has been associated with a favorable prognosis [10**–**12]. TCGA proposed four molecular subgroups based on extensive molecular characterization: Epstein-Barr virus (EBV) positive, microsatellite instable (MSI), genomically stable (GS), and chromosomal instable (CIN) gastric cancer [2]. Patients with an EBV positive or MSI tumor have often been ascribed a favorable overall survival compared to those with an EBV negative or microsatellite stable (MSS) tumor [13**–**15]. TCGA divided EBV negative/MSS gastric cancer into GS and CIN molecular subgroups with an unsupervised clustering algorithm that used the log2 values of specific regions of genome-wide chromosomal copy number aberrations (CNAs), which are calculated by the GISTIC algorithm [2, 16]. Though GS and CIN could clearly be distinguished by clustering, classification criteria to determine if an individual sample belongs to either subtype were not defined. Furthermore, the prognostic value of the GS and CIN subtypes could not be established by TCGA given the limited follow-up data of the cohort.

To approximate TCGA classification alternative methods have been applied [17**–**20]. One of them is the Lauren classification, of which the diffuse type adenocarcinomas are enriched in the GS subgroup. In contrast, the intestinal type tumors have better survival characteristics and are enriched in TCGAs CIN subgroup [2, 17, 21] Also, *CDH1* and *TP53* mutations are enriched in the GS and CIN subgroups, respectively, which has prompted the use of these two genes as surrogates [4, 18, 22, 23]. Finally, detection of allelic imbalances in microsatellites was used to assign tumors into GS or CIN subgroups [20]. This method is an estimate of the proportion of the genome altered.

The lack of a clear definition to assign tumors to either GS and CIN has impeded reproduction in other cohorts making it difficult to establish the biological relevance of chromosomal instability and a clinically relevant association with survival. From a genomic perspective, it could be assumed that the CIN group has worse survival as a high genome instability has been correlated with poor prognosis across tumor types [24**–**31]. However, enrichment of the presumed poor survival CIN subtype for the superior prognosis intestinal subtype does not justify replacement of one binary classification with the other. Similarly, enrichment of the presumed better survival GS subtype for the worse prognosis diffuse subtype does not justify replacement of these classifications either. It does however suggest that a finer grain distinction of EBV negative/MSS gastric cancer is needed.

Well-defined molecular subgroups are important to further unravel the mechanisms underlying gastric cancer development and its heterogeneous clinical behavior. Hence, the aim of the present study is to stratify gastric cancer in a biologically and clinically relevant manner.

## Materials and methods

### Patient material and tumor selection

Formalin-fixed paraffin-embedded (FFPE) resection specimens were collected from patients of the phase III Dutch D1/D2 trial who underwent gastrectomy with curative intent and were randomized between either limited (D1) or more extended (D2) lymphadenectomy without any systemic treatment [32]. Extensive clinical follow-up data of the D1/D2 trial were available.

EBV and MSI status for 467 tumor specimens of the D1/D2 trial (Supplementary Fig. 1) were determined previously [15, 33]. Only EBV negative/MSS tumors (*n* = 371) were included in this study.

Histological tumor type was determined according to Lauren into the two major subtypes intestinal and diffuse type carcinomas on an hematoxylin and eosin (H&E) stained slide by a dedicated gastrointestinal pathologist (NCTvG) [10]. Mixed type tumors were added to the group of diffuse type tumors, assuming that the worst component in terms of differentiation grade determines clinical outcome. Since mucinous carcinomas, carcinomas with lymphoid stroma, and undifferentiated carcinomas, representing only a small proportion of tumors (*n* = 39), do not fit in Lauren’s classification [34], these were excluded from the study. Tumors with insufficient DNA for copy number analyses (*n* = 23) were also excluded. This resulted in a total of 309 tumors included in this study.

The study was conducted in accordance with the Declaration of Helsinki and the Dutch Code of Conduct for Health Research [35]. The study was approved by the Biobank Unit Pathology (BUP2016-71).

### Shallow whole genome sequencing to determine chromosomal copy number aberrations

DNA was extracted as previously described [36]. Briefly, tumor areas were demarcated by NCTvG on a 4 µm glass slide that was H&E stained, serial sections of 10 µm were cut and mounted on glass slides and tumor areas were macrodissected followed by genomic DNA extraction (QIAamp DNA Micro kit, Qiagen, Westburg, Leusden, the Netherlands) [36]. DNA libraries for shallow whole genome sequencing were prepared using TruSeq Nano kits (Illumina, San Diego, CA, USA). Briefly, library preparation consisted of shearing extracted DNA, followed by end repair and 3’ adenylation. Next, we added adapters, enhanced our product by PCR, and then used a single read 50 run on a HiSeq4000 (Illumina). Sequence depth was at least 0.1 x genome coverage. Data analysis was performed in R (version 3.6.1). DNA copy number aberrations (CNA) were extracted by R package QDNAseq (version 1.28.0) that counts the number of sequence reads per bin of 100kbp, followed by blacklisting and corrections of sequencing biases [37]. CN profiles were dewaved by R package NoWaves (version 0.6) [38], and segmented by R package DNAcopy (version 1.66.0) [39]. Tumor cell percentages were estimated using the R package ACE (version 1.10.0) with default settings and a penalty of 0.5 [40]. In case ACE calculated a tumor percentage of 100% as the most likely fit, the second most likely fit was manually chosen. Finally, CNAs were called by R package CGHcall (version 2.54.0) [41] with the tumor percentage as derived from ACE as input variable for the cellularity of each tumor.

### Assignment to TCGA GS/CIN subgroups based on chromosomal copy number aberrations

Publicly available TCGA data of 293 gastric cancer samples were downloaded that included the clinical data with assigned subgroups of these samples as well as their segmented copy number data [2]. In addition, GISTIC 2.0 peaks, chromosomal regions, were downloaded (https://gdc.cancer.gov/about-data/publications/stad_2014) from TCGA that included 31 gains and amplifications and 45 deletions significantly reoccurring in the copy number high group, and 13 gains and amplifications and 25 deletions significantly reoccurring in the copy number low group. TCGA obtained these 114 copy number aberrations by GISTIC 2.0 [16], and used these to classify their samples as GS and CIN by hierarchical clustering [2].

For our analysis with 309 EBV negative/MSS tumors seven peaks on chromosome X were excluded. The log2 copy number value per TCGA sample for each of the remaining 107 reported GISTIC 2.0 peak regions was calculated. The mean log2 value per GISTIC 2.0 peak for all 293 TCGA samples was calculated and combined to obtain an average value for the GS and CIN clusters. For the D1/D2 samples, log2 values were corrected by tumor percentages derived by ACE [40]. Corrected log2 values were calculated for each TCGA GISTIC 2.0 peak for each tumor sample. Tumor samples were assigned to either the GS or CIN clusters based on Euclidean distance, according to TCGAs method to cluster gastric cancers [2]. A heatmap was constructed by R package pheatmap (version 1.0.12).

### Genome instability index calculations based on genome-wide copy number aberrations

CNAs as called by the R package CGHcall were used to calculate the percentage of the genome that was altered, using the genome instability index (GII) previously described [42]. Briefly, CNA calls included −2 for homozygous deletion, −1 for loss, 0 for no CNA, 1 for gain, and 2 for amplification. The total length of the CNAs was divided by the total length of the called genome, and multiplied by 100 to calculate the GII. A cutoff to divide tumors into GII-low and GII-high, based on survival, was set by using the function surv_cutpoint() in package survminer (version 0.4.9) in the program language R (version 4.2.1).

### Immunohistochemistry on CDH1 and p53

Tissue microarrays (TMAs) of *n* = 185 available tumor samples were constructed with three tissue cores per tumor. Immunohistochemical stainings for CDH1 and p53 were performed using the Ventana Benchmark Ultra (Roche, Basel, Switzerland) automated stainer with protein detection by Optiview. Clone NCH-38 by Dako (Agilent, Amstelveen, the Netherlands) was used for CDH1 detection at a dilution of 1/50 and incubated for 32 min after previous treatment of the slides with CC1 for 24 min. CDH1 in 33% or less of tumor cells was labeled “aberrant expression” and more than 33% was considered “normal expression” [22]. Clone DO-7 by Dako was used for p53 detection at a dilution of 1/3000 and incubated for 16 min after prior treatment of the slides with CC1 for 24 min. Both overexpression as well as complete lack of expression of p53 in tumor cells were labeled as “aberrant expression”. Overexpression was considered in case of extra strong staining with a clonal staining pattern. Moderate and variable p53 expression in tumor cells was considered “normal expression”. In case no cells showed any expression and an internal positive control was thus missing, we considered the staining unreliable and excluded these samples from our p53 analyses.

Samples were independently scored by NCTvG and HDB, and discrepant results were discussed until a consensus was reached. The results of CDH1 and p53 IHC were combined into one classification model as previously suggested, resulting in three subgroups: CDH1 aberrant, CDH1 normal and p53 aberrant, and CDH1 normal and p53 normal [22].

### Statistical analysis

Survival analyses were performed on all four classification methods and p-values were calculated by log-rank tests. Cancer-related survival (CRS) was calculated as time from randomization with a median follow-up of 33 months. Kaplan–Meier (KM) plots were right-truncated at 10% of patients per subgroup at risk. All four classifications were included in the multivariable analyses. Only variables known at time of diagnosis were included, since at this time point treatment decisions are being made. Since cTNM stage is notoriously unreliable at time of diagnosis this was excluded for these analyses. Cox regression analyses and stepwise backward selection was used to obtain prognostic relevant subgroups. A *p* < 0.10 was used for this selection. In all other tests a *p* < 0.05 was considered statistical significant. Classification And Regression Tree (CART) analysis was used as second multivariable regression analysis, in which CRS was used as a dependent variable and all four classification methods as independent categorical variables. R packages rpart (version 4.1.15) and rpart.plot (version 3.0.9) were applied for CART analysis. The prognostic relevant subgroups derived from both multivariable analyses were then tested for association with clinicopathological variables by Fisher’s Exact tests. One-way ANOVA was used in case of the continuous variable age. All analyses were conducted using the program language R (version 4.2.1). Frequency plots of most common CNAs were made and compared across subgroups by R packages CGHregions (version 1.40.0) [43] and CGHtest (version 1.1) [44].

## Results

### Tumor selection

After exclusion of 23 tumors with insufficient DNA for copy number analysis, and 39 that were histologically classified as ‘other’ [34], 309 tumors remained available for this study (Supplementary Fig. 1). There was no significant difference in survival between patients who underwent gastrectomy with extended (D2; *n* = 139) compared to limited (D1; *n* = 170) lymphadenectomy (HR 0.96, 95% CI 0.70–1.30, *p* = 0.77; Supplementary Fig. 2).

### Classification of gastric cancer based on chromosomal copy number aberrations

Chromosomal copy number aberrations were generated by shallow whole genome sequencing. Most common gains were of chromosomes 7p, 8, and 20. Gains of chromosome 8 have been reported as most common aberrations in gastric cancer followed by 20 and 7p, which is in line with our data set [45, 46]. Most common losses were of chromosomes 4q, 9p, and 17p, which is also commensurate with previous studies [6, 7, 47]. Assigning samples to the GS and CIN clusters of TCGA resulted in 81 (26.2%) GS and 228 (73.8%) CIN tumors. Alternatively, GII was calculated with all genome-wide CNAs having equal weight. The cutoff that resulted in the largest survival difference between GII-low and GII-high was 14.7%. This cutoff resulted in 73 (23.6%) GII-low and 236 (76.4%) GII-high tumors. The relation between the TCGA GS and CIN subgroups and GII is shown in Supplementary Fig. 3.

### Classification of gastric cancer into subgroups by histopathology or immunohistochemistry

Classification according to Lauren resulted in 143 (45.8%) diffuse or mixed and 166 (54.2%) intestinal type tumors. For IHC of CDH1 and p53 TMAs were constructed with tumor material that was available for 185 tumors. IHC for CDH1 was normal for 159, aberrant for 24, and 2 tumors could not be evaluated. IHC for P53 was normal for 84, aberrant for 89 (23 no expression and 66 overexpression), and 12 tumors could not be evaluated. The combination of CDH1 and p53 resulted in 24 (13.8%) CDH1 aberrant, 81 (46.6%) p53 aberrant, and 69 (39.7%) p53 normal expression tumors.

In the TCGA study CIN tumors were enriched for *TP53* mutations with *TP53* mutations in 71.0% of CIN tumors and in only 14.5% of GS tumors [2]. In this study, we found aberrant p53 expression in 61.1% of CIN tumors and 25.5% of GS. Differences between the proportions of *TP53* mutations in the TCGA cohort and p53 IHC in this study might be explained by the known intratumoral heterogeneous protein expression of p53 [18, 48].

The proportion of tumors with aberrant CDH1 expression (13.1%) in the current 183 tumors with available IHC data is similar to *CDH1* mutations in the 199 (13.1%) tumors with available mutation data in the TCGA cohort [2]. The relation between the TCGA GS and CIN subgroups and the three combined p53/CDH1 subgroups is shown in Supplementary Fig. 4.

### Association of different gastric cancer classification methods with cancer-related survival

For each of these four classification methods Kaplan–Meier analysis was performed with CRS as an end-point. Patients with GS tumors showed a non-significant trend towards longer CRS compared to patients with CIN tumors (HR 0.71, 95% CI 0.49–1.02, *p* = 0.062; Supplementary Fig. 5). Five-year CRS was 57.8% (95% CI 47.6–70.1%) for patients with GS tumors, and 41.6% (95% CI 35.4–49.0%) for patients with CIN tumors. Patients with GII-low tumors showed a non-significant trend towards longer CRS compared to patients with GII-high tumors (HR 0.71, 95% CI 0.49–1.04, *p* = 0.074; Supplementary Fig. 6). Five-year CRS was 57.2% (95% CI 46.7–70.1%) for patients with GII-low tumors, and 42.1% (95% CI 35.9–49.4%) for patients with GII-high tumors. Five-year CRS was 47.9% (95% CI 39.8–57.6%) for patients with GII-low tumors, and 44.6% (95% CI 37.4–53.1%) for patients with GII-high tumors. For Lauren’s classification, patients with intestinal histological type tumors showed a non-significant trend towards longer CRS compared to patients with diffuse/mixed histological type tumors (HR 0.76, 95% CI 0.56–1.03, *p* = 0.077; Supplementary Fig. 7). Five-year CRS was 49.5% (95% CI 42.1–58.3%) for patients with intestinal type tumors, and 42.0% (95% CI 34.3–51.4%) for patients with diffuse type tumors. For p53 and CDH1 IHC, patients with aberrant p53 (HR 1.03, 95% CI 0.56–1.91, *p* = 0.92) or normal p53 (HR 1.16, 95% CI 0.63–2.16, *p* = 0.63) did not have different survival compared to aberrant CDH1 expression tumors (Supplementary Fig. 8). Five-year CRS was 34.1% (95% CI 24.2–48.1%) for patients with p53 normal expression tumors, 40.5% (95% CI 30.5–53.7%) for patients with p53 aberrant expression tumors, and 41.4% (95% CI 25.2–68.1%) for patients with CDH1 aberrant expression tumors.

### Multivariable analysis to determine biological different subgroups by survival

**Fig. 1:**
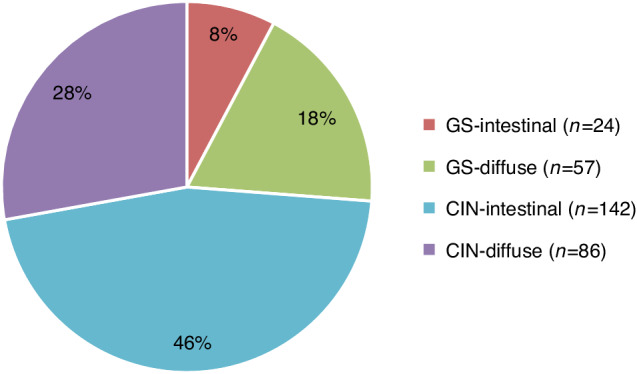
Molecular subgroups within EBV-/MSS gastric cancer in patients from the Dutch D1/D2 trial. Numbers and proportions of the four molecular subgroups are indicated. EBV-/MSS Epstein-Barr virus negative and microsatellite instable, GS genomically stable, CIN chromosomal instable.

Multivariable Cox regression analyses including TCGA, Lauren, GII and p53/CDH1 revealed TCGA’s and Lauren’s classification to be independently associated with longer CRS. This was true for patients with GS (HR 0.60, 95% CI 0.41–0.88, *p* = 0.0087) compared to CIN, and intestinal (HR 0.65, 95% CI 0.47–0.90, *p* = 0.0088) compared to diffuse type tumors in a joined Cox model. CART analysis using TCGA, Lauren, GII and p53/CDH1 revealed a decision tree that first divided tumors TCGA’s classification, and secondly by Lauren in the CIN group only, which resulted in three prognostic relevant subgroups: GS, CIN-intestinal, and CIN-diffuse. The GS subgroup of 81 tumors may have been too small to allow for further distinction into Lauren’s subgroups in this cohort (Supplementary Fig. 9). By combining TCGA’s and Lauren’s classification methods four subgroups can be formed: GS-intestinal (*n* = 24, 7.8%), GS-diffuse (*n* = 57, 18.4%), CIN-intestinal (*n* = 142, 46,0%), and CIN-diffuse (*n* = 86, 27,8%; Fig. 1).

### Clinicopathological characteristics of the GS-intestinal, GS-diffuse, CIN-intestinal, and CIN-diffuse subgroups

**Fig. 2:**
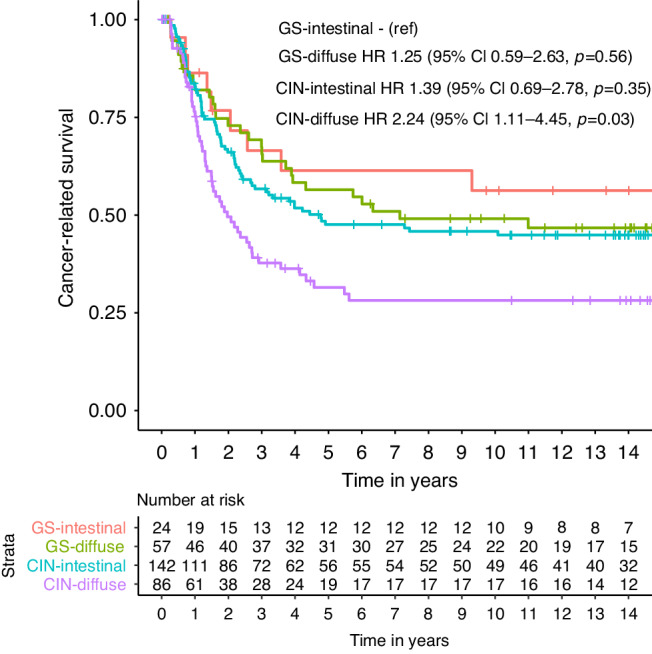
Cancer-related survival in patients with GS-intestinal, GS-diffuse, CIN-intestinal, and CIN-diffuse tumors. Differences in cancer-related survival were assessed using the Kaplan–Meier method and compared using the log-rank test. The hazard ratio was 1.25 (95% CI 0.59–2.63, *p* = 0.56) for GS-diffuse, 1.39 (95% CI 0.69–2.78, *p* = 0.35) for CIN-intestinal, and 2.24 (95% CI 1.11–4.54, *p* = 0.03) for CIN-diffuse, all compared to GS-intestinal. *P* < 0.05 was considered to be statistically significant. GS genomically stable, CIN chromosomal instable.

Patients with GS-diffuse tumors had the youngest age at diagnosis (*p* = 0.028), whereas CIN-intestinal was represented by the oldest patient subgroup (*p* = 0.0029). Only the CIN-intestinal subgroup consisted predominantly of male patients (*p* < 0.001). Diffuse type tumors were more often seen in >2/3 of the stomach compared to intestinal type tumors (*p* = 0.039). GS-intestinal had the highest proportion of pT1 (54.2%, *p* < 0.001), lymph node negative (58.3%, *p* = 0.04), and early stage I (54.2%, *p* = 0.005) tumors, whereas CIN-diffuse had the highest percentage of pT4 (44.2%, *p* < 0.001), lymph node positive (79.1%, *p* = 0.003), and advanced stage IV (8.1%, *p* < 0.001) tumors. CIN is enriched for intestinal (62.3%), and GS for diffuse type (70.4%) tumors, however, the majority of diffuse type tumors are classified as CIN (60.1%). Of the tumors with available IHC data, aberrant CDH1 expression was observed in 10/49 (20.4%) of GS tumors, whereas aberrant p53 makes up 77/126 (61.1%) of CIN tumors. The clinicopathological characteristics of patients with GS-intestinal, GS-diffuse, CIN-intestinal, and CIN-diffuse tumors are shown in Table 1. Five-year CRS was 61.4% (95% CI 43.5–86.6%) for patients with GS-intestinal tumors, 56.5% (95% CI 44.8–71.2%) for GS-diffuse, 47.6% (95% CI 39.7–57.1%) for CIN-intestinal, and 31.5% (95% CI 22.5–44.1%) for CIN-diffuse (Fig. 2).

**Table 1:** Clinicopathological characteristics of patients with GS, CIN-intestinal, and CIN-diffuse/mixed tumors.

	GS-intestinal	GS-diffuse	CIN-intestinal	CIN-diffuse	*P* value
	(*n* = 24)	(*n* = 57)	(*n* = 142)	(*n* = 86)	
Age					0.019
Mean (range)	63.0 (42–83)	60.9 (33–80)	65.9 (21–84)	62.6 (31–84)	
Sex					0.0023
Male	9 (37.5%)	23 (40.4%)	91 (64.1%)	40 (46.5%)	
Female	15 (62.5%)	34 (59.6%)	51 (35.9%)	46 (53.5%)	
Tumor localization					0.24
Proximal	4 (16.7%)	3 (5.3%)	15 (10.6%)	8 (9.3%)	
Middle	8 (33.3%)	16 (28.1%)	36 (25.4%)	21 (24.4%)	
Distal	11 (45.8%)	29 (50.9%)	80 (56.3%)	41 (47.7%)	
>2/3 of stomach	1 (4.2%)	9 (15.8%)	11 (7.7%)	16 (18.6%)	
pT stage					0.00050
pT1	13 (54.2%)	17 (29.8%)	24 (16.9%)	5 (5.8%)	
pT2	1 (4.2%)	6 (10.5%)	20 (14.1%)	11 (12.8%)	
pT3	6 (25.0%)	19 (33.3%)	62 (43.7%)	31 (36.0%)	
pT4	4 (16.7%)	15 (26.3%)	36 (25.4%)	38 (44.2%)	
Missings	*0 (0.0%)*	*0 (0.0%)*	*0 (0.0%)*	*1 (1.2%)*	
pN stage					0.0045
pN0	14 (58.3%)	23 (40.4%)	44 (31.0%)	18 (20.9%)	
pN1	3 (12.5%)	7 (12.3%)	32 (22.5%)	16 (18.6%)	
pN2	2 (8.3%)	14 (24.6%)	39 (27.5%)	18 (20.9%)	
pN3	5 (20.8%)	13 (22.8%)	27 (19.0%)	34 (39.5%)	
TNM (7th edition)					0.0010
Stage I	13 (54.2%)	17 (29.8%)	33 (23.2%)	9 (10.5%)	
Stage II	4 (16.7%)	15 (26.3%)	39 (27.5%)	20 (23.3%)	
Stage III	6 (25.0%)	24 (42.1%)	67 (47.2%)	50 (58.1%)	
Stage IV	1 (4.2%)	1 (1.8%)	3 (2.1%)	7 (8.1%)	
p53 IHC					0.00011
Normal expression	8 (33.3%)	27 (47.4%)	25 (17.6%)	24 (27.6%)	
Aberrant expression	5 (20.8%)	7 (12.3%)	48 (33.8%)	29 (33.7%)	
Missings	*11 (45.8%)*	*23 (40.4%)*	*69 (48.6%)*	*33 (38.4%)*	
CDH1 IHC					0.092
Normal expression	11 (45.8%)	28 (49.1%)	71 (50.0%)	49 (57.0%)	
Aberrant expression	3 (12.5%)	7 (12.3%)	5 (3.5%)	9 (10.5%)	
Missings	*10 (41.7%)*	*22 (38.6%)*	*66 (46.5%)*	*28 (32.6%)*	
Genome Instability Index					<0.0001
GII-low	15 (62.5%)	45 (78.9%)	5 (3.5%)	8 (9.3%)	
GII-high	9 (37.5%)	12 (21.1%)	137 (96.5%)	78 (90.7%)	

Data are presented as number (percentage) of patients unless otherwise indicated.

*GS* genomically stable, *CIN* chromosomal instable, *IHC* immunohistochemistry, *GII* Genome Instability Index.

### Chromosomal copy number aberrations in the four prognostic subgroups

**Fig. 3:**
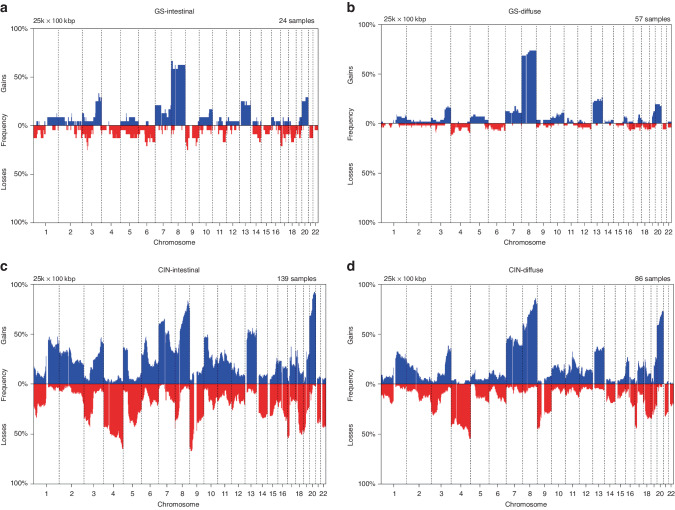
Frequency plots of copy number aberrations per subgroup. Frequency plots of copy number aberrations in (**a**) GS-intestinal, (**b**) GS-diffuse, (**c**) CIN-intestinal, and (**d**) CIN-diffuse tumors. The frequency of gains are depicted in blue and losses in red. GS genomically stable, CIN chromosomal instable.

Frequency plots of copy number gains and losses are depicted for GS-intestinal, GS-diffuse, CIN-intestinal, and CIN-diffuse tumors, separately (Fig. 3a–d). As expected, GS tumors harbored only few copy number aberrations with most frequently gains of chromosome 8. Chromosomal aberrations of GS-intestinal and GS-diffuse tumors were similar with only a few marginally statistically significant (*p* < 0.05; FDR < 0.27) differences between these two subgroups: losses of chromosomes 4p16.3, 6q14.1–q15, and 10p12.31–p12.1 occurred more often in GS-intestinal than GS-diffuse tumors, whereas gains of chromosome 8 occurred more often in GS-diffuse than GS-intestinal tumors. Called aberrations occurred more often in CIN-intestinal than CIN-diffuse tumors and most statistically significant (*p* < 0.001; FDR < 0.1) were losses of chromosomes 1p36.13–p35.2, 5q13.2–q31.2, 8p, 9p21.3–p21.1, 10q23.2–q25.1, 14q21.1–q22.1, 15q25.1–q25.2, 16q23.1–q24.2, and gains of chromosomes 2q12.3–q36.1, 5p, 6p25.1–q14.2, 10p–q22.3, 10q26.13, 11p14.3–p13, 15q25.3–q26.3, 20q. Gains of chromosome 8p occurred more often in CIN-diffuse than CIN-intestinal tumors.

**Fig. 4:**
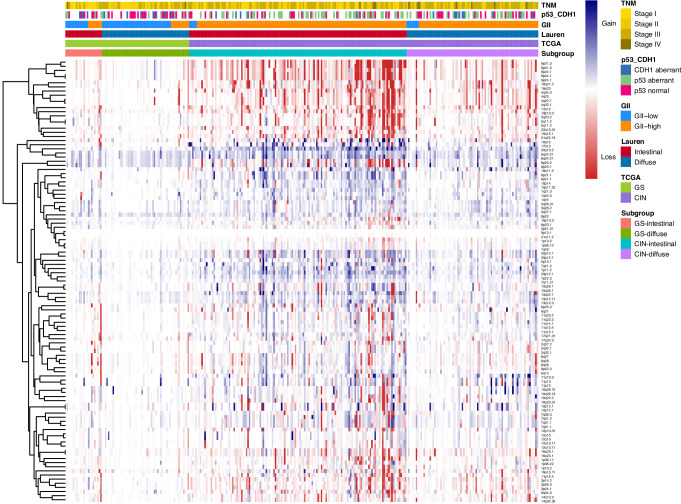
Heatmap of copy number aberrations per GISTIC peak for each patient from the Dutch D1/D2 trial. Copy number aberrations are depicted on the y-axis and unsupervised clustered by GISTIC peaks. Copy number gains are depicted in blue and losses in red. Patients are depicted on the x-axis and not clustered, but only grouped for each of the four proposed subgroups GS-intestinal, GS-diffuse, CIN-intestinal, and CIN-diffuse. GS genomically stable, CIN chromosomal instable, GII genome instability index, p53_CDH1, combined p53 and CDH1 classification.

Figure 4 shows a heat map of the copy number aberrations of all GISTIC peaks clustered together for each tumor with its clinicopathological characteristics.

## Discussion

Multiple efforts have been made to establish biological and clinical relevant subgroups for the vast majority of gastric cancer, hence EBV negative/MSS gastric cancer classified as GS or CIN by TCGA [2, 10, 19, 22]. This study demonstrates that the most relevant chromosomal copy number aberrations as proposed by TCGA can be used to assign samples to the GS and CIN subgroups. In addition, this study shows that GS and CIN have significant prognostic value in addition to the established Lauren’s classification, whereby patients with CIN tumors having worse CRS compared to GS tumors [2].

To the best of our knowledge, this is the first time that TCGA’s gastric cancer GS and CIN chromosomal copy number-based subgroups were reproduced in an independent cohort. Classification of 309 tumors based on the specific chromosomal locations determined by TCGA resulted in similar proportions of GS (26.2%) and CIN (73.8%) as identified for the TCGA cohort (28.3% and 71.7%, respectively) [2].

We used two different types of multivariable regression analyses to establish prognostic and clinical relevant subgroups. First, multivariable Cox regression analyses revealed TCGA’s and Lauren’s classifications as only classification methods to be independently associated CRS. CART analyses confirmed both TCGA’s classification and Lauren’s classification as independent biological factors that impact survival. It was also shown that both TCGA and Lauren’s classification are important in stratifying gastric cancer samples based on survival.

We identified two entities of diffuse as well as intestinal type tumors, GS-intestinal and GS-diffuse as well as CIN-intestinal and CIN-diffuse, that have distinct survival in gastric cancer. Although diffuse type tumors are generally thought to have poor prognosis [49, 50], we show that a substantial part of the diffuse type tumors classified as GS (57/143; 40.0%), is associated with longer CRS compared to CIN-diffuse tumors. In literature GS tumors are thought to be associated with a lower copy number load and therefore also with good prognosis [2, 26, 27]. Likewise, intestinal tumors are thought to have longer survival compared to diffuse type tumors [17, 21]. Our data show that stratifying gastric cancer by Lauren’s classification alone ignores the clinically distinct copy number based subgroups within intestinal and diffuse type tumors.

The proposed molecular/histological subgroups show correlations with pT and pN stages. Table 1 shows that the GS-intestinal subgroup is associated with lowest pT and pN stages, while the CIN-diffuse subgroup has the highest proportion of pT4 and pN3 stages. In addition, 7 out of 12 patients that were diagnosed with metastatic (peritoneal) disease in the surgical resection specimen were of the CIN-diffuse subgroup. Clinicians should be aware of the high risk of clinically undetected metastases, especially in this subgroup. The association between subgroups and pTNM stage substantiates the idea that these subgroups may represent better or worse biological behavior.

GS and CIN subgroups differ in the mean number of chromosomal copy number alterations. We therefore included the proportion of chromosomal regions altered across the genome (GII) in our analyses. A similar approach was used by Kohlruss et al., who used allelic imbalances in 22 microsatellite markers across the genome to estimate the proportion of the genome altered [20]. However, multivariate analyses showed that not GII, but only alterations in the specific chromosomal regions that were associated with CIN by TCGA are independently associated with survival. This finding suggests that some chromosomal regions contribute more to the clinical behavior of tumors than other regions.

Correlation between p53 IHC and *TP53* mutation status has been reported, however, p53 expression has also been reported not to be able to reliably predict *TP53* mutation status in individual cases [18, 51]. In our study, we did not find an association between p53 IHC and CRS. This could explain that a substantial proportion of tumors would be misclassified if *TP53* status is used as an alternative for chromosomal instability. Even though the proportion of tumors with aberrant CDH1 expression by IHC in our study was similar to those with *CDH1* mutations in the TCGA cohort, true GS cases will be missed by using CDH1 as an alternative marker, since only 20/54 (37.0%) GS tumors in TCGA harbored a *CDH1* mutation [2]. In addition, the CDH1 IHC scoring system will remain subjective and lacks a general cutoff.

As far as we are aware, this is the first study that shows the prognostic value of copy number-based GS and CIN tumors in a large cohort of resectable gastric cancers with long clinical follow-up. None of the patients received perioperative systemic treatment, which implies that these observations mirror the biology of the tumor and its prognosis. A limitation of this study is the number of samples available for CDH1 (183/309, 59.2%) and p53 (173/309, 56.0%) IHC, caused by the limited amount of remaining tumor tissues.

Clinical implementation of TCGA classification is hampered by the fact that it did not provide a method to classify individual tumors into GS or CIN. This was due to the fact that they used clustering analyses of a large series of gastric cancer to build their classification. Our method allows to classify individual gastric carcinomas based on their chromosomal abnormalities into GS and CIN subtypes, and paves the way to evaluate its clinical impact. Our study concerned resectable gastric carcinomas treated with surgical resection. This has the advantage to study patient outcome in the different subgroups without interference of multimodality treatment, thus reflecting differences in biological behavior of the tumor. The current standard of care however consists of perioperative chemotherapy and surgical resection [52]. Our method paves the way for future studies to evaluate if this classification has also predictive value for response to either established or new treatment regimens. This could be done in retrospective or prospective analyses of well-defined patient cohorts. Also, the distinction in GS and CIN could be considered to take along as stratification marker in clinical trials, similar to Lauren’s classification. Only when therapeutic decisions depend on this classification it may be considered for implementation in diagnostic practice.

In conclusion, based on multiple classification methods, we identified four subgroups in EBV-/MSS gastric cancer with different biological characteristics and distinct prognosis: GS-intestinal, GS-diffuse, CIN-intestinal, and CIN-diffuse. Remarkably, patients with diffuse type tumors and a GS genotype have significantly better survival than their CIN counterparts. These four prognostically distinct subgroups can be determined prior to treatment and may have clinical value in the stratification of patients for future clinical trials.

## Supplementary information

Supplementary information

## Data Availability

Sequence data has been deposited at the European Genome-phenome Archive (EGA), which is hosted by the EBI and the CRG, under accession number EGAS00001007394.

## Code availability

The code for assignment of tumors to GS or CIN subgroups is available upon request through the corresponding author.
