# Supplementary material for: Chromosomal copy number based stratification of gastric cancer has added prognostic value to Lauren’s histological classification
Source: BJC Rep. 2024 Aug 19;2:58. doi: 10.1038/s44276-024-00078-2 (PMC11523994; doi:10.1038/s44276-024-00078-2)
Supplement: Supplementary file 1 — Supplementary information [file 44276_2024_78_MOESM1_ESM.pdf]

Supplementary Figure 1. Flow of patients in the study.

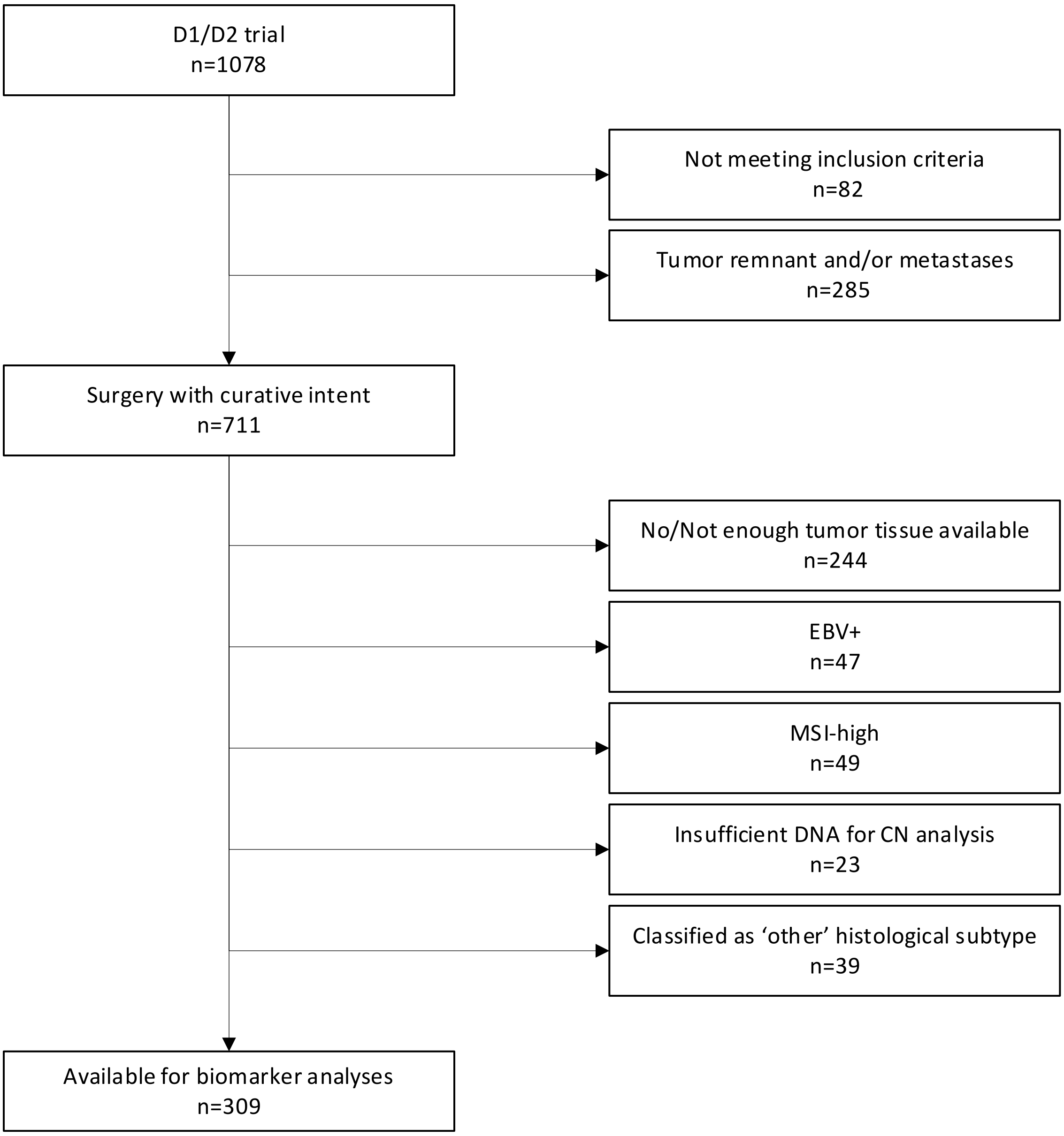

Abbreviations: EBV, Epstein-Barr virus; MSI, microsatellite instability; EBV+, EBV positive; MSI-high, microsatellite instable; EBV-/MSS, EBV negative and microsatellite stable; CN, copy number.

Supplementary Figure 2. Cancer-related survival in the patients who underwent gastrectomy with limited (D1) or extended (D2) lymphadenectomy in the Dutch D1/D2 trial.

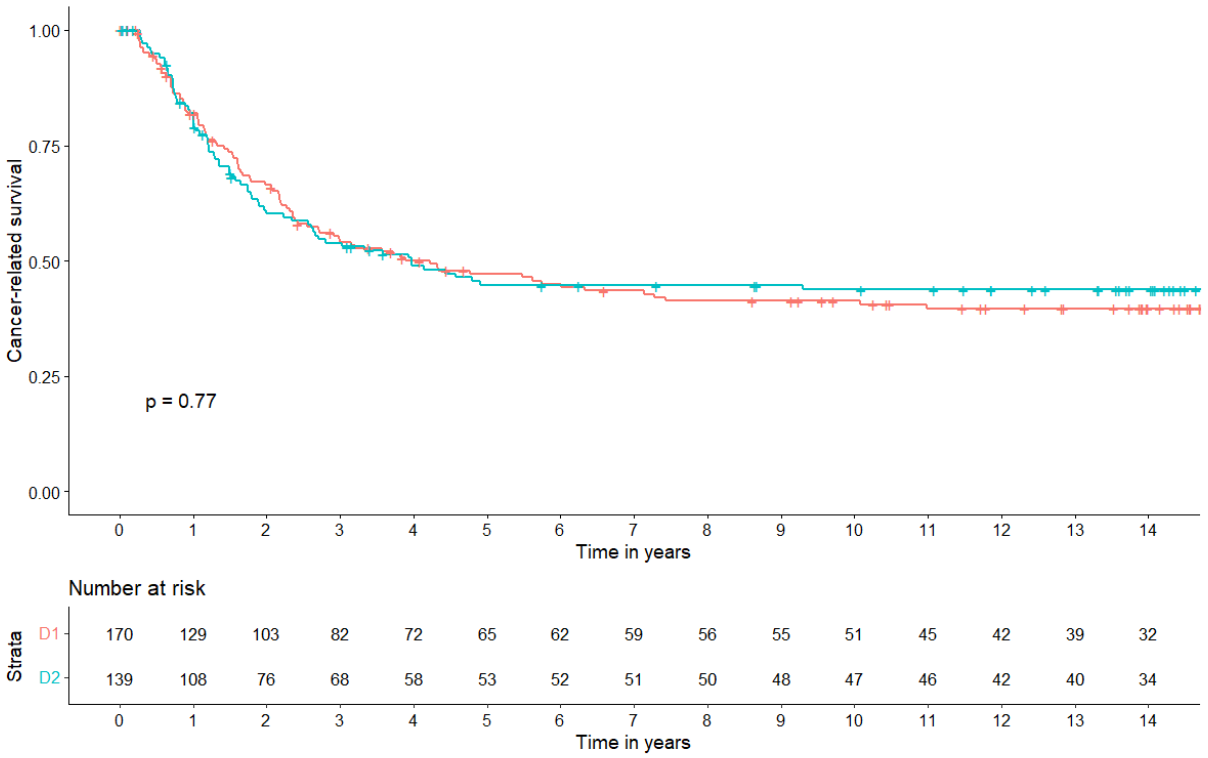

Differences in cancer-related survival were assessed using the Kaplan-Meier method and compared using the log-rank test. The hazard ratio was 1.05 (95% CI 0.77-1.43, p=0.77) for patients who underwent gastrectomy with limited (D1) compared to extended (D2) lymphadenectomy. P<0.05 was considered to be statistically significant.

Five-year cancer-related survival was 47.1% (95% CI 39.8-55.7%) for patients who underwent gastrectomy with limited (D1) lymphadenectomy, and 44.7% (95% CI 36.8-54.3%) for patients who underwent gastrectomy with extended (D2) lymphadenectomy.

Supplementary Figure 3. Bar plot of GII with GS/CIN

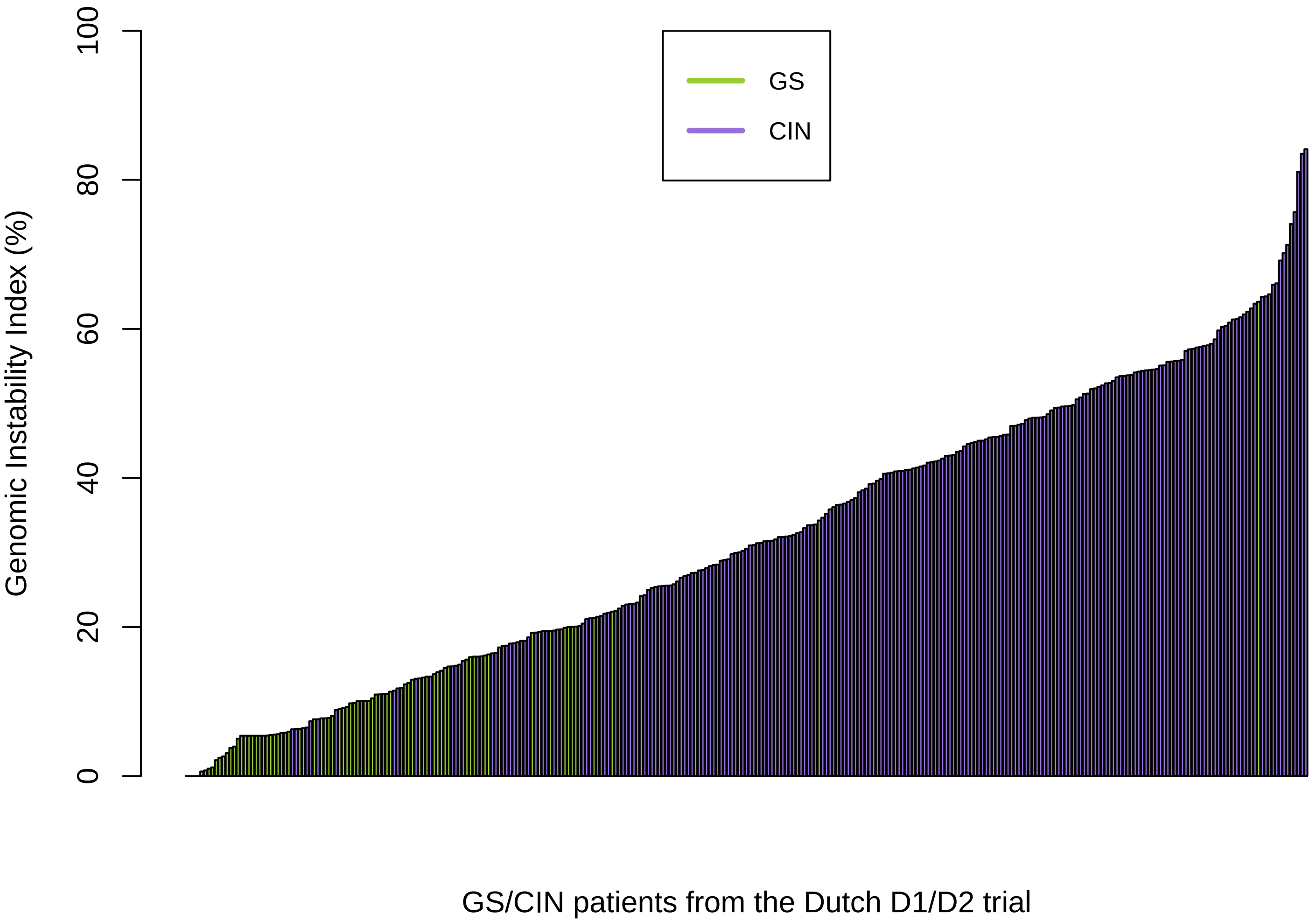

Supplementary Figure 4. Sunburst chart of overlapping classifications

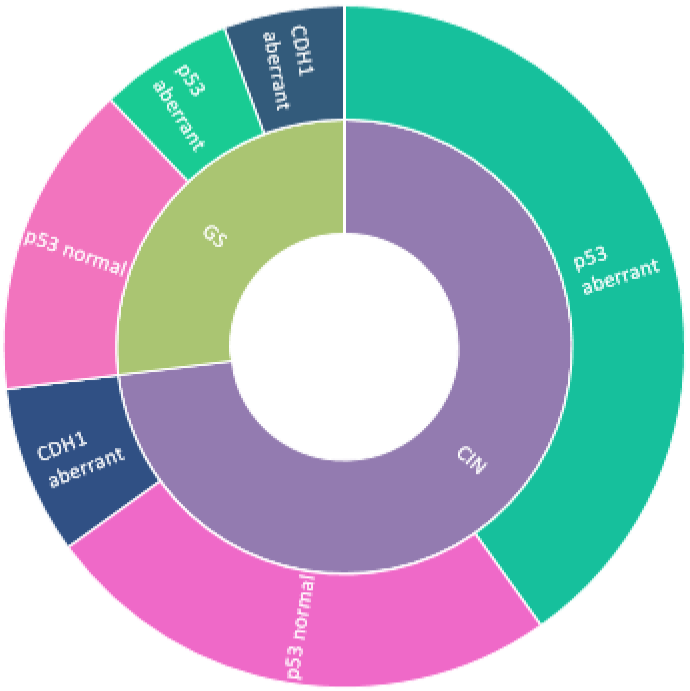

Overlap of the GS/CIN classification proposed by TCGA and the CDH1/p53 classification proposed by Setia et al.[1]

Abbreviations: GS, genomically stable; CIN, chromosomal instable; p53 aberrant, normal CDH1 and aberrant p53 expression by immunohistochemistry; p53 normal, normal CDH1 and normal p53 expression by immunohistochemistry; CDH1 aberrant, aberrant CDH1 expression by immunohistochemistry.

1. Setia N, Agoston AT, Han HS, et al. A protein and mRNA expression-based classification of gastric cancer. *Mod Pathol* 2016; **29**(7): 772-84.

Supplementary Figure 5. Cancer-related survival in the patients with GS and CIN tumors in the Dutch D1/D2 trial.

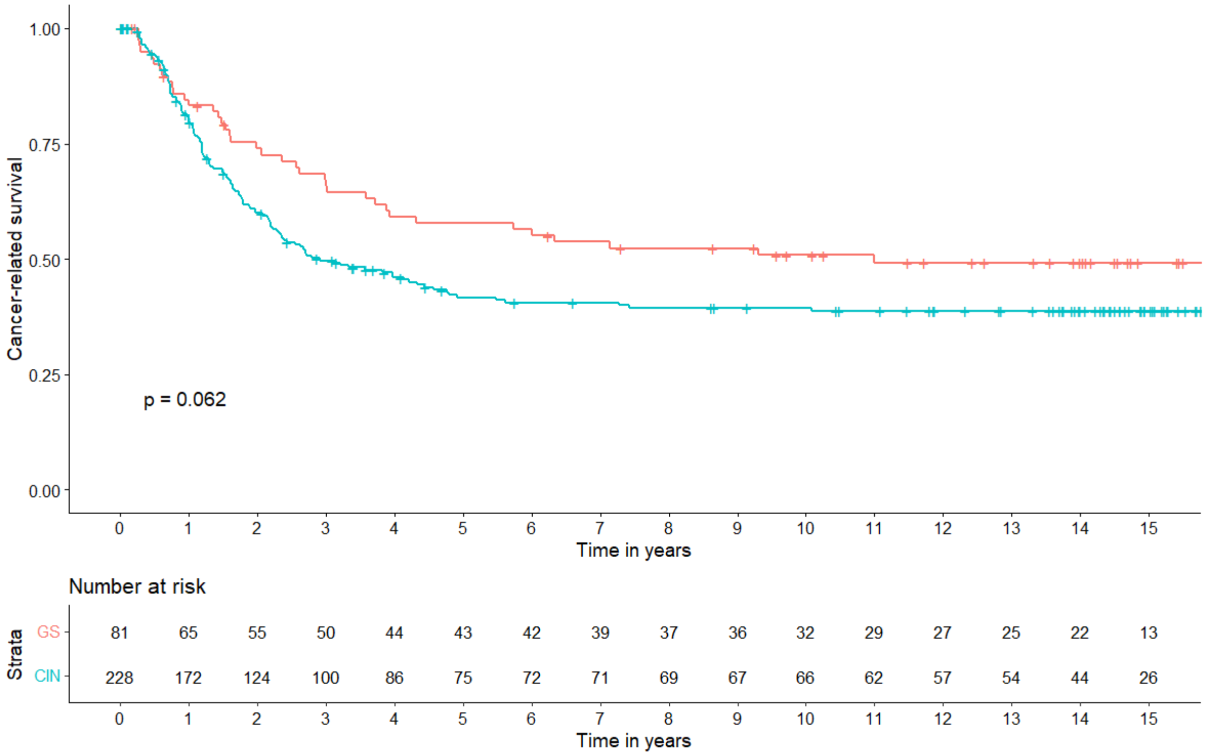

Differences in cancer-related survival were assessed using the Kaplan-Meier method and compared using the log-rank test. The hazard ratio was 0.71 (95% CI 0.49-1.02, p=0.062) for patients with GS compared to CIN tumors. P<0.05 was considered to be statistically significant.

Five-year cancer-related survival was 57.8% (95% CI 47.6-70.1%) for patients with GS tumors, and 41.6% (95% CI 35.4-49.0%) for patients with CIN tumors.

Abbreviations: GS, genomically stable; CIN, chromosomal instable

Supplementary Figure 6. Cancer-related survival in the patients with GII-low and GII-high tumors in the Dutch D1/D2 trial.

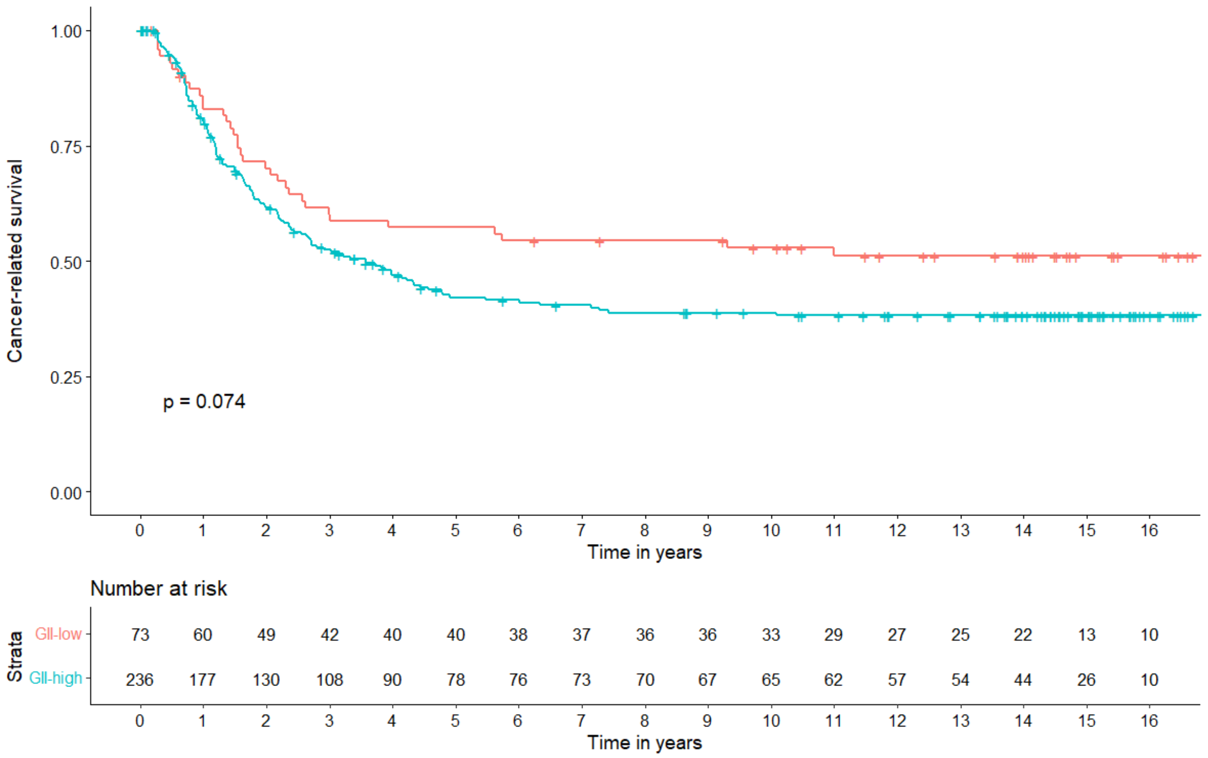

Differences in cancer-related survival were assessed using the Kaplan-Meier method and compared using the log-rank test. The hazard ratio was 0.71 (95% CI 0.49-1.04, p=0.074) for patients with GII-low compared to GII-high tumors. P<0.05 was considered to be statistically significant.

Five-year cancer-related survival was 57.2% (95% CI 46.7-70.1%) for patients with GII-low tumors, and 42.1% (95% CI 35.9-49.4%) for patients with GII-high tumors.

Abbreviations: GII, genome instability index

Supplementary Figure 7. Cancer-related survival in the patients with intestinal and diffuse type tumors in the Dutch D1/D2 trial.

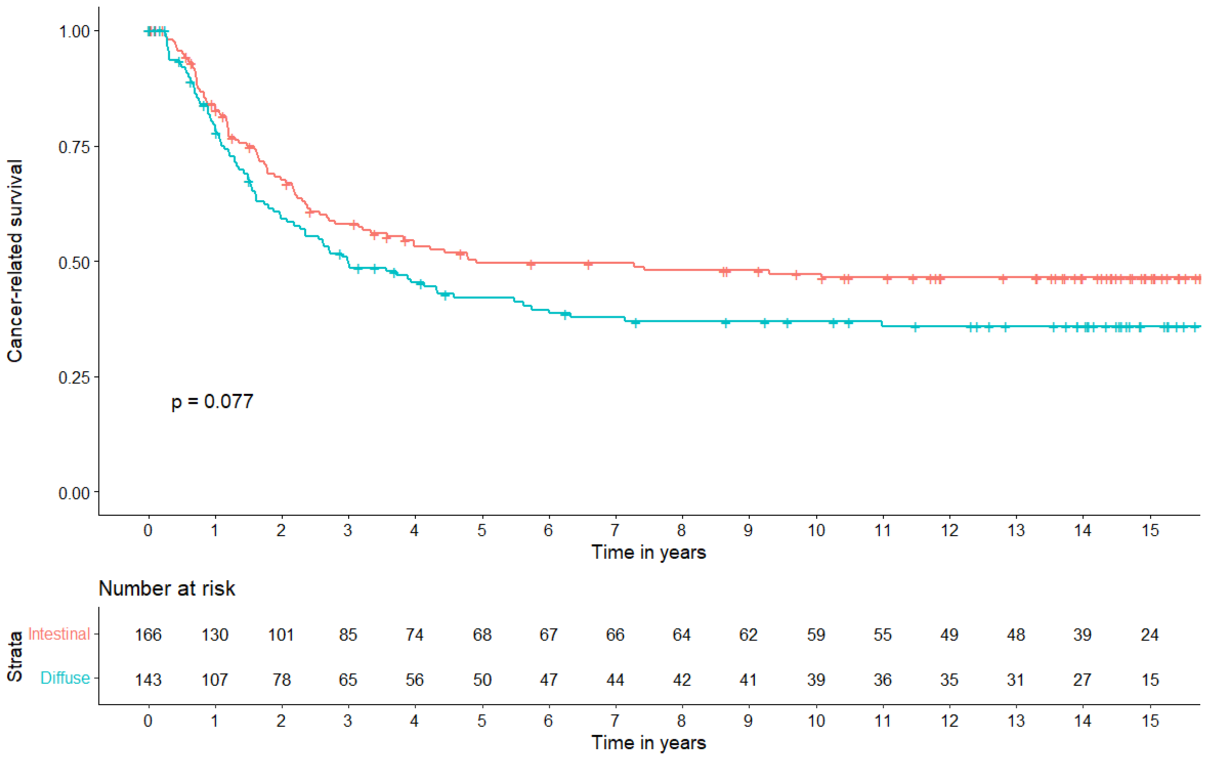

Differences in cancer-related survival were assessed using the Kaplan-Meier method and compared using the log-rank test. The hazard ratio was 0.76 (95% CI 0.56-1.03, p=0.077) for patients with intestinal compared to diffuse type tumors. P<0.05 was considered to be statistically significant.

Five-year cancer-related survival was 49.5% (95% CI 42.1-58.3%) for patients with intestinal type tumors, and 42.0% (95% CI 34.3-51.4%) for patients with diffuse type tumors.

Supplementary Figure 8. Cancer-related survival in the patients with E-cadherin aberrant, p53 aberrant and p53 normal IHC expression tumors in the Dutch D1/D2 trial.

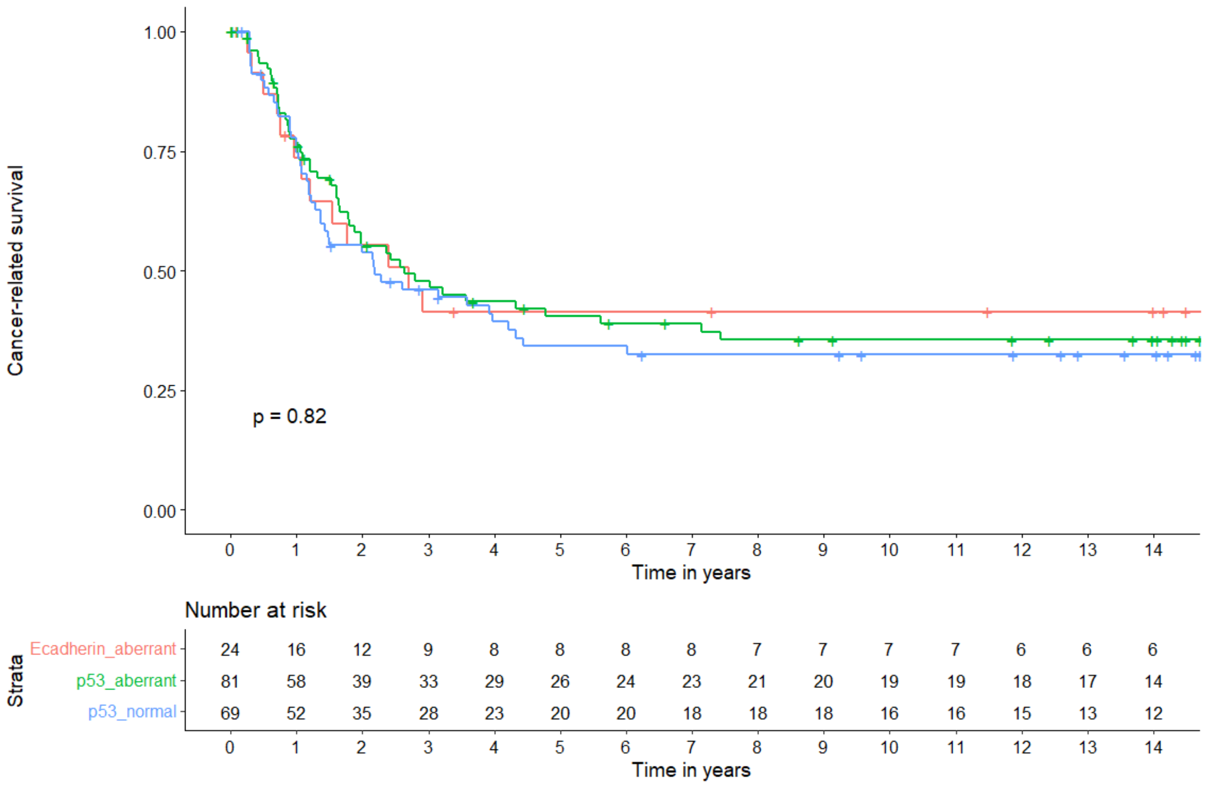

Differences in cancer-related survival were assessed using the Kaplan-Meier method and compared using the log-rank test. The hazard ratio was 1.03 (95% CI 0.56-1.91, p=0.92) for patients with aberrant p53 and 1.16 (95% CI 0.63-2.16, p=0.63) for normal p53 compared to aberrant E-cadherin expression tumors.

Five-year cancer-related survival was 34.1% (95% CI 24.2-48.1%) for patients with p53 normal expression tumors, 40.5% (95% CI 30.5-53.7%) for patients with p53 aberrant expression tumors, and 41.4% (95% CI 25.2-68.1%) for patients with CDH1 aberrant expression tumors.

Abbreviations: IHC, immunohistochemistry

Supplementary Figure 9. Output of CART to determine prognostically distinct subgroups in EBV-/MSS gastric cancer in 309 patients in the Dutch D1/D2 trial.

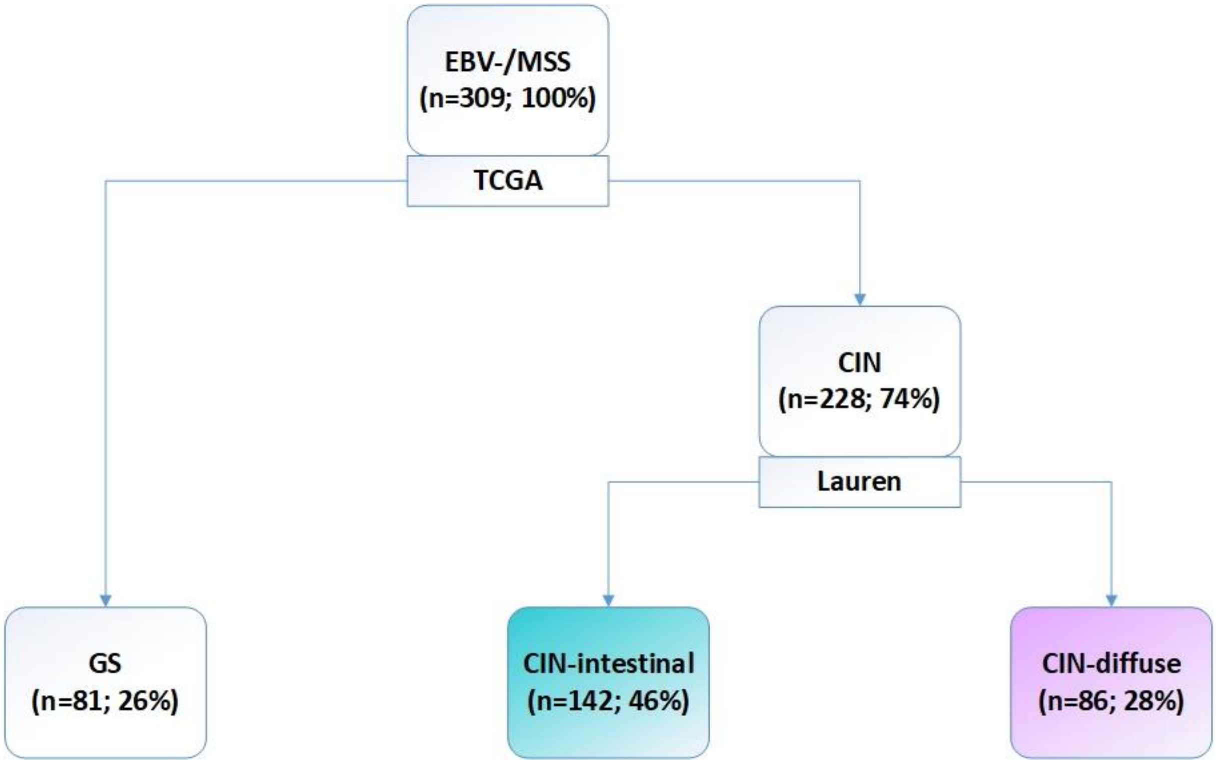

Input variables are cancer-related survival as dependent variable, and the independent variables TCGA (GS or CIN), genome instability index (GII; high or low), (iii) Lauren's classification (diffuse or intestinal), and (iv) CDH1/p53 immunohistochemistry (IHC; aberrant CDH1, normal-, or aberrant p53 expression).

Abbreviations: CART, classification and regression tree; EBV-, Epstein-Barr virus negative; MSS, microsatellite stable; TCGA, The Cancer Genome Atlas; GS, genomically stable; CIN, chromosomal instable; GII, genome instability index; IHC, immunohistochemistry.
